# Endometrial microbiota is more diverse in people with endometriosis than symptomatic controls

**DOI:** 10.1038/s41598-021-98380-3

**Published:** 2021-09-23

**Authors:** Jocelyn M. Wessels, Miguel A. Domínguez, Nicholas A. Leyland, Sanjay K. Agarwal, Warren G. Foster

**Affiliations:** 1 Department of Obstetrics & Gynaecology, McMaster University, Hamilton, ON L8S 4K1 Canada; 2 Facultad de Medicina Veterinaria y Zootecnia, Universidad Autónoma de Tamaulipas, 87000 Cd. Victoria, TAMPS Mexico; 3 Department of Reproductive Medicine, University of California San Diego, La Jolla, CA 92037 USA

**Keywords:** Reproductive disorders, Microbiome

## Abstract

Endometriosis is a chronic, estrogen-dependent gynecological condition affecting approximately 10% of reproductive age women. The most widely accepted theory of its etiology includes retrograde menstruation. Recent reports suggest the uterus is not sterile. Thus, the refluxed menstrual effluent may carry bacteria, and contribute to inflammation, the establishment and growth of endometriotic lesions. Here, we compared and contrasted uterine bacteria (endometrial microbiota) in people with surgically confirmed presence (N = 12) or absence of endometriosis (N = 9) using next-generation 16S rRNA gene sequencing. We obtained an average of > 9000 sequence reads per endometrial biopsy, and found the endometrial microbiota of people with endometriosis was more diverse (greater Shannon Diversity Index and proportion of ‘Other’ taxa) than symptomatic controls (with pelvic pain, surgically confirmed absence of endometriosis; diagnosed with other benign gynecological conditions). The relative abundance of bacterial taxa enriched in the endometrial microbiota of people with endometriosis belonged to the Actinobacteria phylum (Gram-positive), *Oxalobacteraceae* (Gram-negative) and *Streptococcaceae* (Gram-positive) families, and *Tepidimonas* (Gram-negative) genus, while those enriched in the symptomatic controls belonged to the *Burkholderiaceae* (Gram-negative) family, and *Ralstonia* (Gram-negative) genus. Taken together, results suggest the endometrial microbiota is perturbed in people with endometriosis.

## Introduction

Endometriosis is a complex disease that affects approximately 10% of women of reproductive age, and often associated with the main clinical features of pelvic pain (mechanisms involved in pain and endometriosis reviewed in^1^) and infertility. It is caused by development of endometrial-like glands and stroma outside the uterus, and though its exact pathogenesis remains unclear, it appears to involve a combination of contributing factors such as retrograde menstruation into the peritoneal cavity^2^, and an altered immune response^3,4^. Other theories on the origin of endometriotic lesions include the embryonic rest theory (prenatal endometrial precursor cells differentiate and become established in the pelvic region)^5–7^, coelomic metaplasia (transformation of the peritoneal mesothelium), lymphovascular metastasis (transportation of endometrial cells via lymphatics or blood), or endometrial stem/progenitor cells (reviewed in^8–10^). However, other factors (anatomical, genetic, environmental, lifestyle, menstrual cycle dynamics, aberrant immune responses, etc.)^10–14^ are likely involved as 90% of women experience retrograde menstruation^15^, but only about 10% develop endometriosis^13^. Recent studies have also suggested serum metabolites are altered in endometriosis^16^, that genetic/epigenetic changes caused by retrograde menstruation into the peritoneal cavity contribute to lesion development (reviewed in^17^), and that genetic pre-disposition may differ in relation to ethnicity^18^.

Even though the human body is home to more than ten times more bacteria than nucleated human cells (ratio is 1:1 if compared with all cells in the body, due to many non-nucleated red blood cells)^19,20^, for years the uterus was thought to be sterile and not contain bacteria. This was likely because bacterial culture was the main technique employed to identify bacteria. However, many bacterial species are difficult, if not impossible, to grow in vitro due to nutritional or other environmental requirements. Nevertheless, a few bacterial culture studies in females undergoing in vitro fertilization (IVF) hinted at the existence of an endometrial (uterine) microbiota when the presence of bacteria on embryo transfer catheters was negatively associated with IVF success^21–23^. With the advent of advanced molecular biology techniques like 16S rRNA gene sequencing, the identification of microorganisms in the microbiotas collected from different sites of the body has become fairly routine. Indeed, even the long-held belief that the uterine environment was sterile has been challenged with next generation sequencing reports of microbial signatures in the uterus^24–33^. However, because these studies mainly included fertility patients or those with gynecological disease, there is some uncertainty surrounding the endometrial microbiota of “healthy” women. Some of the presently contested points include (1) the source of endometrial microbiota seeding, (2) the existence of a stable, resident endometrial microbiota in the “healthy” state, (3) the bacterial species included in a normal/”healthy” endometrial microbiota (if it exists), and (4) whether uterine bacteria are transient and only associated with pathologies (reviewed in^34^). Nevertheless, it appears as though bacteria participate in uterine-related diseases like endometriosis^26,31,33^, endometrial cancer^35,36^, and uterine fibroids^30^. In fact, reports of bacterial endotoxin in the pelvic cavity and menstrual blood of people with endometriosis^37^ have led to a bacterial contamination hypothesis suggesting endotoxin/bacteria in the menstrual effluent contributes to pelvic inflammation, growth, and progression of endometriotic lesions (reviewed in^38,39^). Furthermore, several studies suggest the microbiotas (gut, vaginal, cervical, uterine/endometrial) of patients with endometriosis differ females without this condition (reviewed in^40^). Therefore, the objective of the present study was to compare the endometrial microbiota recovered from endometrial biopsies of patients with surgically confirmed presence or absence of endometriosis (cases versus symptomatic controls—patients with pain but without endometriosis). We chose to include symptomatic controls, rather than asymptomatic controls, because of the aforementioned uncertainty surrounding the endometrial microbiota of “healthy” women, and because one of our aims was to examine differentially expressed taxa that might be unique to patients with endometriosis as compared to other gynecological conditions.

## Materials and methods

### Study participants

The study was approved by the Research Ethics Board, McMaster University (Institutional Review Board no. 06-064, 14-066-T), and all participants provided written informed consent and basic demographic/gynecological history prior to participation. All methods were performed under the approved study protocol, in accordance with the relevant guidelines and regulations. In this prospective, cross-sectional study 24 patients attending McMaster University Medical Centre were selected for the present study from a larger study on endometriosis (2011–2017). This study size was determined based on our previous work in the vaginal microbiota where differences in the microbiota could be observed with a group size of ten^41^. A sample size calculation was also performed. We anticipate a difference between groups of approximately 1.2 on the Shannon Diversity Index, and a standard deviation of 0.8, based on our previous study^41^. A sample size calculation using a two-tailed t-test to achieve a power of 80% and alpha of 5%, with a SD = 0.8 and expected difference of 1.2 between groups, indicates that 9 women per study group would be required for the present study. Patients at our tertiary care centre were undergoing gynecological laparoscopy for pelvic pain thought to be due to endometriosis. During surgery patients were categorized as a case or symptomatic control (people with pain but no surgical or pathological evidence of endometriosis) by the gynecological surgeon (NAL), and diagnoses were confirmed by histopathology. Of the 24 patients recruited, 14 were diagnosed with endometriosis (Cases; Stage 1: 0, Stage 2: 1, Stage 3: 1, Stage 4: 12) using the revised American Fertility Score (rAFS)^42^, while 10 were diagnosed with other benign gynecological conditions (Symptomatic Controls). Exclusion criteria were people unable to provide consent, aged under 18, currently pregnant, or who had used hormone therapies (oral contraceptives, GnRH agonist/antagonist, progestins, etc.) in the 3 months preceding study enrollment.

### Endometrial biopsy collection

Immediately before surgery the vagina was swabbed with chlorhexidine in preparation for the gynecological laparoscopy. A sterilized vaginal speculum was inserted, and then a double sheathed, sterile pipelle endometrial suction curette (Cooper Surgical, Trumbull, CT, USA) was passed through the cervix to collect an endometrial biopsy, taking care to avoid contact with the vaginal wall and cervix. Biopsies were deposited in sterile 15 mL Falcon conical tubes (polystyrene) (Fisher Scientific, Ottawa, ON, Canada) and transported to the laboratory on ice, where they were processed within 30 min. One portion of the endometrial biopsy was fixed in 10% buffered formalin (Staplex Scientific, Etobicoke, ON, Canada) and processed for routine histology. Slides were cut for each biopsy in 5 µm sections, and stained with hematoxylin and eosin to confirm menstrual cycle phase using the Noyes criteria^43^. A second portion of the endometrial biopsy was placed in RNAlater (Sigma-Aldrich Canada, Oakville, ON, Canada), kept at 4 °C overnight, and then stored at − 80 °C until processed for nucleic acid extraction.

### Bacterial V3 region of 16S rRNA gene sequencing

Biopsies were thawed, weighed (10–50 mg) and homogenized in 700 µL Qiazol lysis buffer (Qiagen, Hilden, Germany) using a Pro200 tissue homogeniser (PRO Scientific, Oxford, CT, USA). Total nucleic acid extraction was performed using the RNeasy Mini Kit (Qiagen, Hilden, Germany), omitting the DNase treatment, and following the manufacturer’s protocol. The quantity and purity of nucleic acids was assessed using the Nanodrop 2000 (Thermo Fisher Scientific, Burlington, ON, Canada). A final volume of 80 µL was frozen and stored at − 80 °C until required for sequencing. Samples were numerically coded and researchers were blinded to experimental groups until data analysis.

To retain bacterial DNA and eliminate RNA that might inhibit the PCR reaction, RNase A (Qiagen Hilden, Germany) was added to the first PCR mastermix. The hypervariable V3 region of the 16S rRNA gene was amplified using a two-stage (nested) PCR approach. Initially the 8f. (AGAGTTTGATCCTGGCTCAG) to 1492r (CACGGATCCTACGGGTACCTTGTTACGACTT) region of the 16S rRNA gene was amplified in triplicate using 100–200 ng of DNA template with 2U of Taq, 1 × buffer, 1.5 mM MgCl_2_, 0.4 mg/mL BSA, 0.2 mM dNTPs, 50 µg/mL RNaseA, and 10pmols of each primer. The initial PCR reaction was carried out at 94 °C for 5 min, 15 cycles of 94 °C for 30 s, 56 °C for 30 s and 72 °C for 90 s, with a final extension of 72 °C for 10 min. The triplicate reaction was then combined and used as the template in the second stage of the nested PCR. In the second PCR, 3 µL of the first PCR reaction product was used as the template and was combined with 2U of Taq, 1 × buffer, 1.5 mM MgCl_2_, 0.4 mg/mL BSA, 0.2 mM dNTPs, and 5pmols each of Illumina adapted primers 341F (CCTACGGGAGGCAGCAG) and 518R (ATTACCGCGGCTGCTGG) (primers + Illumina adapters/barcode/priming region as described in supplemental materials of Bartram et al., 2011: ~ 80 bp)^44–46^. The PCR reaction was carried out at 94 °C for 5 min, 35 cycles of 94 °C for 30 s, 50 °C for 30 s, and 72 °C for 30 s, with a final extension of 72 °C for 10 min. Each PCR run contained no template negative controls (sterile water), which did not yield PCR products (no 300 bp band on agarose gel). Resulting PCR products were visualized on a 1.5% agarose gel. Positive amplicons (visualization of a 300 bp 16S band on the agarose gel) were normalized using the SequalPrep normalization kit (Thermo Fisher Scientific A1051001), and resultant PCR products were sequenced by the McMaster Genomics Facility (Hamilton, ON), using the Illumina MiSeq platform. The resulting 16S sequences were processed as previously described, by sl1p, our in-house data pipeline^45^. As per the McMaster Genomics Facility protocol, samples not yielding a PCR product for the 16S rRNA gene were not sent for sequencing. These samples were considered to be negative. A representative 1.5% agarose gel demonstrating the presence or absence of a PCR product following the two-stage PCR for the 16S rRNA gene in our endometrial samples is presented in Supplemental Fig. 1. There were 3 endometrial biopsies (2 Cases, and 1 Control) that did not yield a PCR product for the 16S rRNA gene and were thus considered negative (the band at ~ 80 bp represents dimers of primers + Illumina adapters/barcode/priming region). Therefore, subsequent analyses were performed on the remaining 12 Cases and 9 Controls.

Alpha-diversity excluding singletons was calculated using the sl1p pipeline^45^, and QIIME version 1.9.1-dev. Ten rarefaction tables with 3848 sequences were used. Observed species, Chao1, and Shannon Diversity were graphed and analyzed using GraphPad Prism (GraphPad Software Inc., La Jolla, CA). Linear discriminant analysis (LDA) effect size (LEfSe)^47^ (https://huttenhower.sph.harvard.edu/galaxy/) was used to determine if there were significant taxonomic differences in the endometrial microbiota of cases and symptomatic controls. Alpha values of 0.05, and the 2.0 threshold for logarithmic LDA score for discriminative features were selected for LDA analysis. Taxa bar charts, Bray–Curtis dissimilarity PCoAs, the gap statistic, and heatmaps were generated in R version 3.2.3 (R Core Team, 2015) as described^46^. For species level estimations (described in^46^) in the heatmap, most of the OTUs could not be resolved to the species level (100% identity and coverage on NCBI’s nucleotide BLAST: https://blast.ncbi.nlm.nih.gov/Blast.cgi using the 16S rRNA database), and are thus reported as the resultant genera from our 16S sequencing.

### Statistical analysis

Researchers were blinded to experimental groups until data analysis. Demographic characteristics were tested for normality and groups were statistically compared using SigmaPlot (SigmaPlot 10.0, Systat Software Inc., San Jose, CA, USA). Two-tailed Student’s t-tests (for age of the study participants, age at menarche, years since menarche, and duration of menstrual bleeding), Fisher’s exact tests (for smoking status), and Chi square (for ethnicity, occupational status, and menstrual cycle stage) were used to compare Cases and Controls. A p value ≤ 0.05 was considered significant for all statistical tests employed.

The 16S data was tested for normality and alpha-diversity metrics were statistically compared using multiple unpaired t-tests, corrected for multiple comparisons using the Holm–Sidak method (GraphPad Software Inc., La Jolla, CA). Beta-diversity was assessed between groups by permutational multivariate analysis of variance (PERMANOVA), using Bray–Curtis dissimilarity distance matrices and employing the adonis function in the vegan package^48^ in R. Data are presented as mean ± SEM, unless otherwise indicated.

## Results

### Study participants

The characteristics describing the study participants included in this report are shown in Supplemental Table 1. Mean age, ethnicity, occupational status, smoking status, number of years since menarche, duration of menstrual bleeding, and stage of the menstrual cycle at surgery were similar between symptomatic controls and patients affected by endometriosis. However, symptomatic controls did report a significantly earlier age at menarche (11.6 ± 1.0 vs. 13.0 ± 1.3 years, *p* = 0.015).

### Endometrial microbiota

Three endometrial biopsies (2 Cases, and 1 Symptomatic Control) did not yield a PCR product for the 16S rRNA gene and were thus considered negative. Therefore, 16S rRNA gene analyses were performed on the remaining 12 Cases and 9 Symptomatic Controls. The minimum number of 16S sequences read during sequencing was 867, and the maximum was 20,113. The average number of sequence reads for all study participants was 9155.6 ± 1613.8.

### Diversity of the endometrial microbiota of people with endometriosis is greater than in symptomatic controls

**Figure 1:**
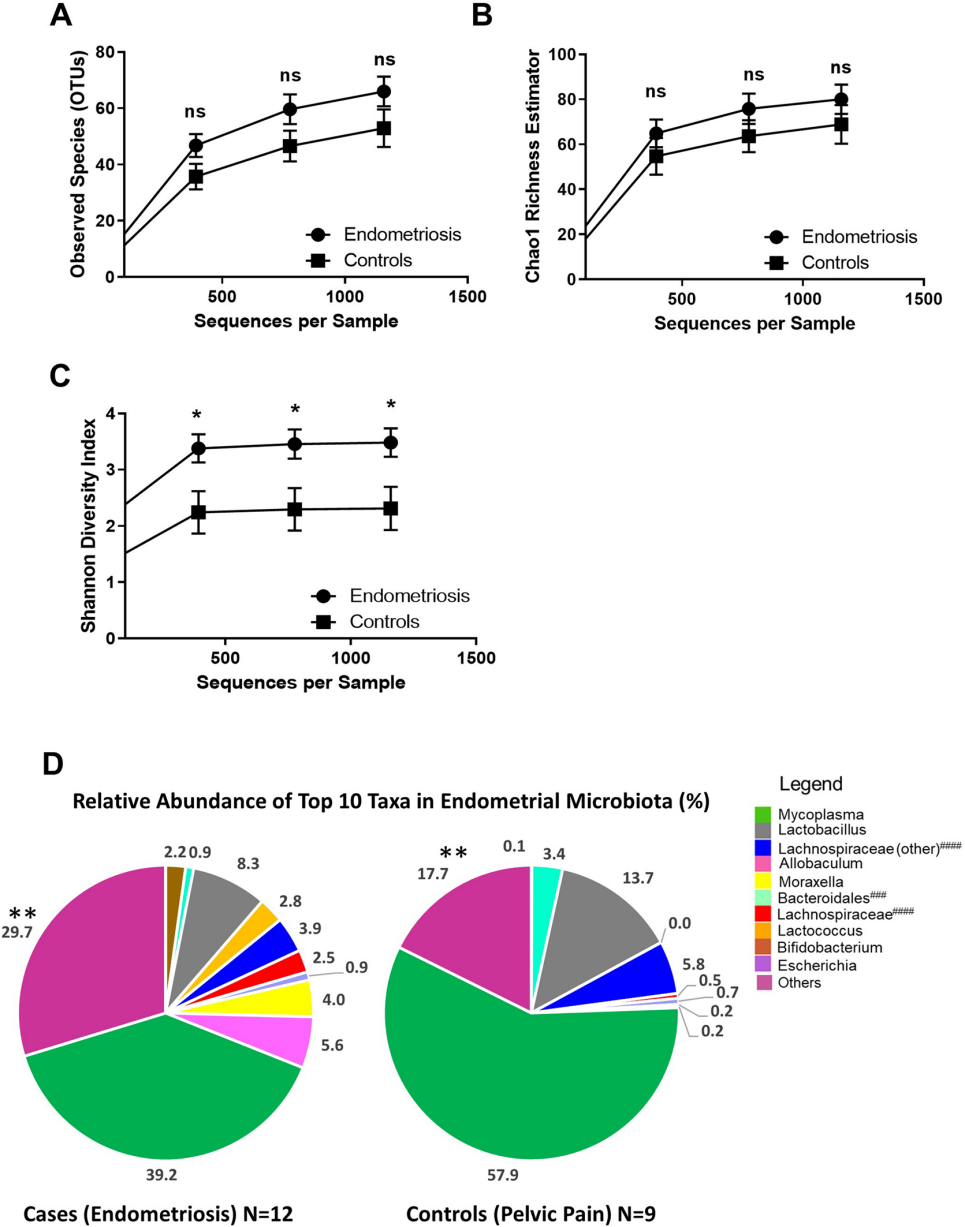
Endometrial microbiota of patients with endometriosis is significantly more diverse than symptomatic controls. Three alpha-diversity metrics were used to compare bacterial richness and evenness within the endometrial microbiota of patients with surgically confirmed endometriosis (N = 12) and surgically confirmed controls (with pelvic pain, but not endometriosis, N = 9). (**A**) No significant differences in Observed Species or (**B**) Chao 1 Richness were observed at the levels of rarefaction where graphs levelled off and the greatest number of samples was retained (multiple unpaired t-tests, corrected for multiple comparisons using the Holm–Sidak method). (**C**) Patients with endometriosis had significantly greater bacterial diversity as assessed by the Shannon Diversity Index at all levels of rarefaction where the graph levelled off and the greatest number of samples was retained (adjusted *p* ≤ 0.05; multiple unpaired t-tests, corrected for multiple comparisons using the Holm–Sidak method). (**D**) The average percent relative abundance of the top 10 taxa in the endometrial microbiota of cases and controls was plotted as a pie chart and the proportion of taxa assigned to the ‘Others’ category was significantly greater in patients with endometriosis than symptomatic controls (unadjusted *p* = 0.007; adjusted *p* = 0.07, Mann–Whitney U test without/with correction for multiple comparisons using the Holm–Sidak method). *p ≤ 0.05, **p ≤ 0.01. Data is presented as mean ± SEM (**A–C**) and as a percentage (**D**). Ns: not significant. OTUs: operational taxonomic units. ###: resolved to bacterial order, ####: resolved to bacterial family.

First, we sought to compare bacterial richness and evenness in the endometrial microbiota of patients with surgically confirmed endometriosis (N = 12) versus surgically confirmed symptomatic controls (N = 9; people with pelvic pain, but not endometriosis). Three alpha-diversity (estimates of species diversity within the endometrial microbiota of an individual) metrics were used to compare the endometrial microbiota of these groups (Fig. 1). No significant differences in Observed Species (Fig. 1A) or Chao 1 Richness (Fig. 1B) were observed at the levels of rarefaction where graphs levelled off and the greatest number of samples was retained (multiple unpaired t-tests, corrected for multiple comparisons using the Holm–Sidak method). However, people with endometriosis had significantly greater bacterial diversity as assessed by the Shannon Diversity Index at all levels of rarefaction (Fig. 1C) (adjusted *p* ≤ 0.05; multiple unpaired t-tests, corrected for multiple comparisons using the Holm–Sidak method). We also plotted the average percent relative abundance of the top 10 taxa in the endometrial microbiota of cases and controls, and found the proportion of taxa assigned to the ‘Others’ category was significantly greater in patients with endometriosis than symptomatic controls (Fig. 1D) (29.7 ± 3.1% in Cases vs. 17.7 ± 2.9% in Controls; unadjusted *p* = 0.007, Mann–Whitney U Test). However, after adjusting for multiple comparisons (Holm–Sidak method), the relationship verged on significance (adjusted *p* = 0.07). No significant differences between the other top 10 taxa were observed.

When we repeated these analyses only including people with stage 4 endometriosis (N = 10) versus controls (N = 9) (Supplemental Fig. 2), we saw the same results as above (no significant difference in Observed Species or Chao 1 (multiple unpaired t-tests, corrected for multiple comparisons using the Holm–Sidak method), and a greater significant difference in Shannon Diversity Index at all levels of rarefaction (adjusted *p* ≤ 0.01; multiple unpaired t-tests, corrected for multiple comparisons using the Holm–Sidak method)). We also saw the proportion of taxa assigned to the ‘Others’ category was significantly greater in patients with stage 4 endometriosis than symptomatic controls (31.3 ± 3.5% in Stage 4 Cases vs. 17.7 ± 2.9% in Controls; unadjusted *p* = 0.008, Mann–Whitney U Test). However, after adjusting for multiple comparison (Holm–Sidak method), the relationship verged on significance (adjusted *p* = 0.08).

### Beta-diversity of the endometrial microbiota of people with and without endometriosis

**Figure 2:**
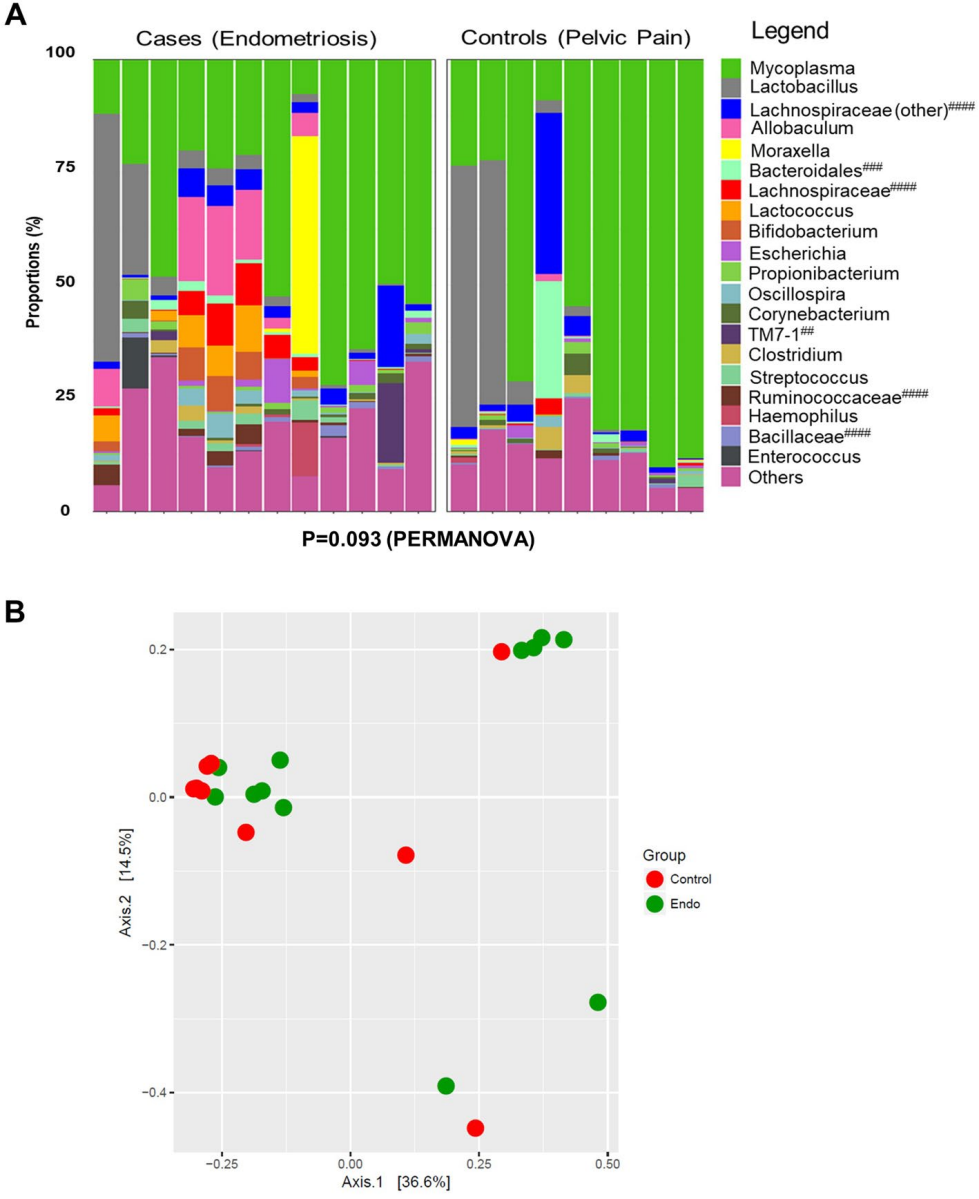
Beta-diversity of patients with endometriosis verges on being significantly greater than symptomatic controls. (**A**) The top 20 bacterial genera in the endometrial microbiota were plotted by relative abundance as taxa bar charts and compared between patients with surgically confirmed endometriosis (N = 12) and surgically confirmed controls (N = 9). Each bar represents the endometrial microbiota of one individual. Each colour represents a different genus of bacteria, as indicated in the legend. Endometrial microbiota are ordered left to right in descending order of the relative abundance of lactobacilli. Patients with endometriosis (as a group) had an endometrial flora that verged on being significantly different from the endometrial microbiota of surgically confirmed, symptomatic controls (β-diversity, *p* = 0.09, PERMANOVA). ##: Resolved to bacterial class. ###: Resolved to bacterial order. ####: Resolved to bacterial family. (**B**) The principal coordinate analysis plots (PCoA) demonstrated the beta-diversity of the endometrial microbiota at the OTU level based on the Bray–Curtis dissimilarity matrix (endometriosis = green dots, controls = red dots). Axes = eigenvalues, a metric whose magnitude indicates the amount of variation captured in the PCoA axis.

**Figure 3:**
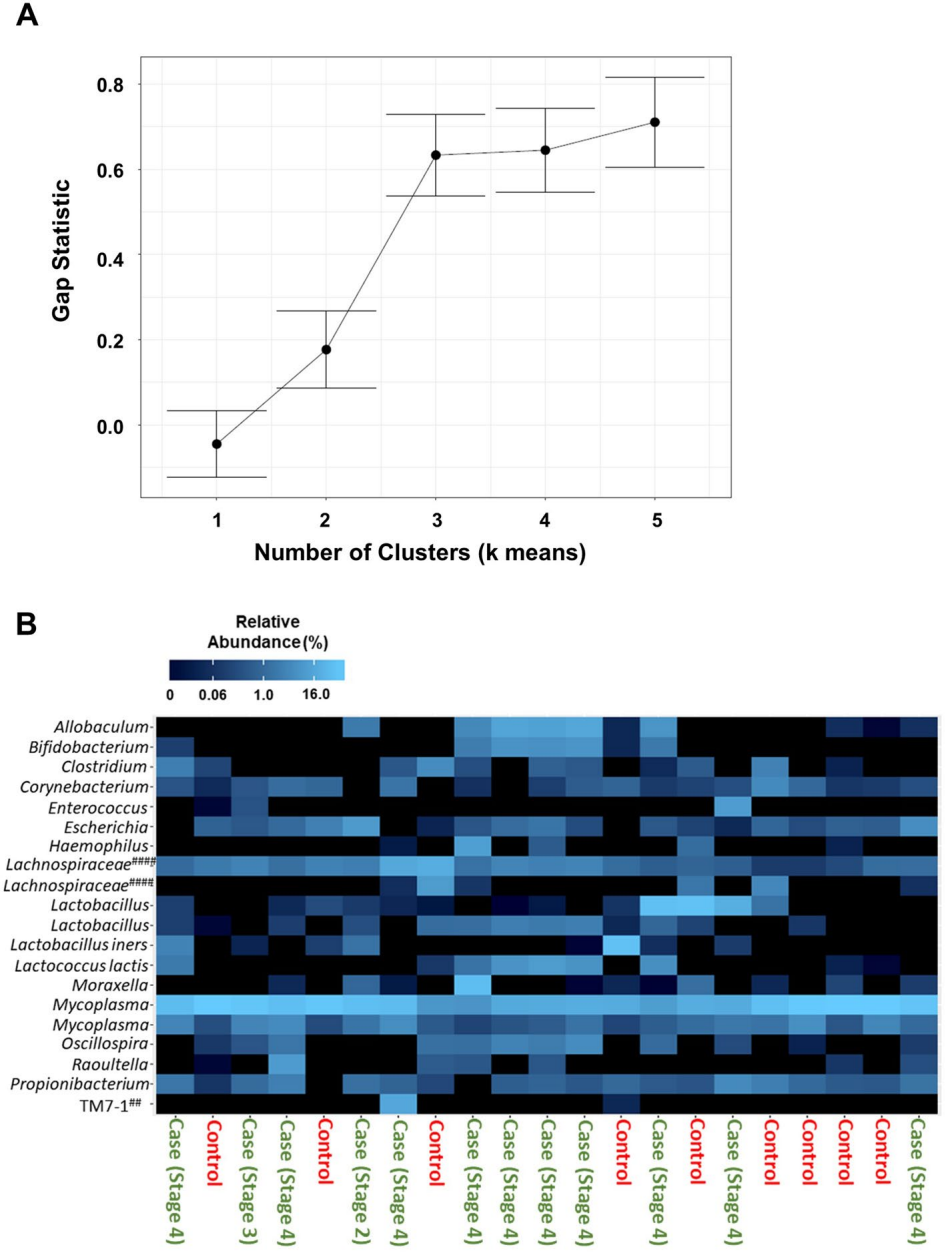
Examination of endometrial microbiota clustering from patients with endometriosis and symptomatic controls. (**A**) The gap statistic was calculated to give an estimation of the number of clusters found in the PCoA. Three clusters were present in the data, as indicated by the plateau in the gap statistic, which occurred at a value of 3 in this graph. (**B**) A heatmap of the top 20 bacterial taxa based on Bray–Curtis dissimilarity distance and PCoA ordination demonstrated the endometrial microbiota (columns) by disease status (Endometriosis Cases vs. Symptomatic Controls). Taxa are ordered alphabetically along the y-axis. ##: Resolved to bacterial class. ####: Resolved to bacterial family.

Next, we examined beta-diversity (estimates of species diversity within the endometrial microbiota of one group versus another). The top 20 bacterial genera in the endometrial microbiota were plotted by relative abundance as individual taxa bar charts (Fig. 2A) and compared between patients with surgically confirmed endometriosis (N = 12) and surgically confirmed symptomatic controls (N = 9). Each bar represents the endometrial microbiota of one person. Each colour represents a different genus of bacteria, as indicated in the legend. Endometrial microbiota are ordered left to right in descending order of the relative abundance of lactobacilli. Patients with endometriosis had endometrial flora that verged on being significantly different from the endometrial microbiota of surgically confirmed, symptomatic controls (β-diversity, *p* = 0.09, PERMANOVA). We also plotted a principal coordinate analysis plot (PCoA) to demonstrate the beta-diversity of the endometrial microbiota at the OTU level based on the Bray–Curtis dissimilarity matrix (Fig. 2B). Endometrial microbiota did not appear to cluster by disease status (endometriosis vs. controls) during principal coordinates analysis, however three clusters were identified in the data using the gap statistic (K-means clustering) (Fig. 3A). PCoA ordination and the Bray–Curtis dissimilarity distance were used to construct a heatmap (Fig. 3B).

### Taxonomic differences in the endometrial microbiota of cases and symptomatic controls

**Figure 4:**
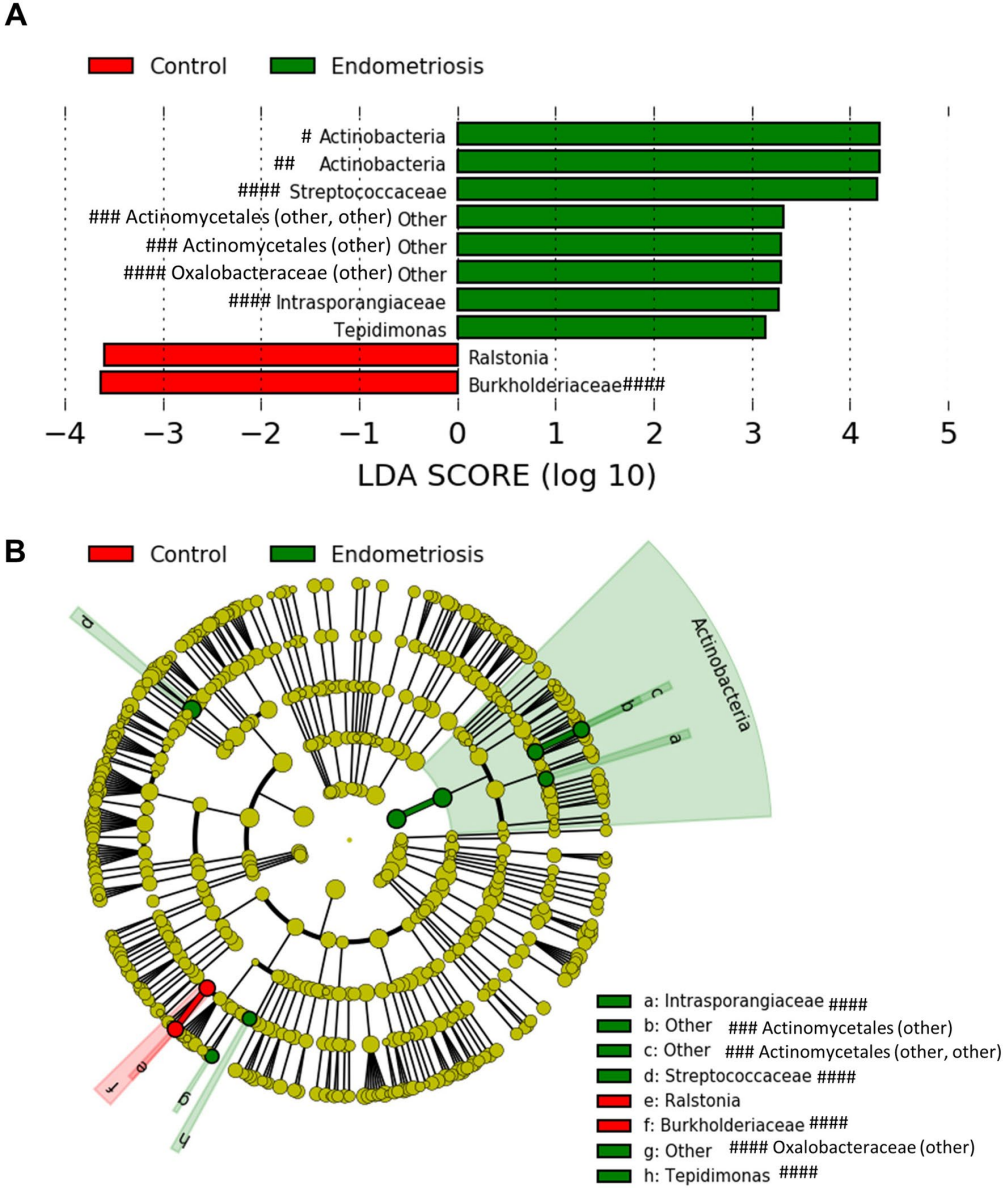
Differential bacterial taxa in the endometrial microbiota of patients with endometriosis versus symptomatic controls. (**A**) Linear Discriminant Analysis effect size (LefSe) analysis separated the endometrial microbiota of patients with endometriosis from control women with pelvic pain based on relative abundance of the bacterial genera listed, using 2.0 as a threshold for discriminative features, and *p* ≤ 0.05 for statistical tests. LEfSe analysis revealed enrichment of taxa including bacteria in the Actinobacteria phylum, *Oxalobacteraceae* and *Streptococcaceae* families, and *Tepidimonas* genus in patients with endometriosis, while symptomatic controls were found to have enrichment of the *Burkholderiaceae* family, and *Ralstonia* genus. (**B**) Cladogram of differential taxa in the endometrial microbiota of patients with endometriosis (in terms of relative abundance—enriched taxa in green) and controls with pelvic pain, but without endometriosis (enriched taxa in red). #: Resolved to bacterial phylum. ##: Resolved to bacterial class. ###: Resolved to bacterial order. ####: Resolved to bacterial family.

Finally, we wanted to determine which bacterial taxa were differentially represented (in terms of relative abundance) in the endometrial microbiota of patients with endometriosis versus symptomatic controls. We performed a LefSe analysis that separated the endometrial microbiota of cases from symptomatic controls based on relative abundance of the bacterial genera listed (Fig. 4A). Our LEfSe analysis revealed enrichment of taxa including bacteria in the Actinobacteria phylum, *Oxalobacteraceae* and *Streptococcaceae* families, and *Tepidimonas* genus in patients with endometriosis, while symptomatic controls had enrichment of the *Burkholderiaceae* family, and *Ralstonia* genus. A cladogram was created to show the relationship between bacterial taxa and highlight the differential taxa (in terms of relative abundance) in the endometrial microbiota of patients with endometriosis and symptomatic controls (Fig. 4B).

## Discussion

Herein, we compared the endometrial microbiota recovered from endometrial biopsies of patients with surgically confirmed presence or absence of endometriosis (cases versus symptomatic controls—patients with pain but without endometriosis). We demonstrate the presence of an endometrial microbiota in the uterus of patients with endometriosis that is more diverse (greater Shannon Diversity Index (an estimator of species richness and evenness^49^), and greater proportion of ‘Other’ taxa) than that of symptomatic controls (patients with pelvic pain, but surgical absence of endometriosis). This suggests although the total number of bacterial species in the endometrial microbiotas is similar (Observed Species and Chao 1 Richness were comparable between groups), the species evenness differs between Cases and Controls. In other words, there is more variability in species abundance in Cases as compared to Controls^49^. Although the effect of enhanced diversity of the endometrial microbiota in people with endometriosis is presently unknown, perhaps this variability differentially induces an immune response, ultimately contributing to disease pathophysiology. The relative abundance of bacterial taxa enriched in the endometrial microbiota of patients with endometriosis belonged to the Actinobacteria phylum (Gram-positive), *Oxalobacteraceae* (Gram-negative) and *Streptococcaceae* (Gram-positive) families, and *Tepidimonas* (Gram-negative) genus, while those enriched in the symptomatic controls belonged to the *Burkholderiaceae* (Gram-negative) family, and *Ralstonia* (Gram-negative) genus. Our results align with a recent systematic review^50^ that found 9 of 15 studies profiling the microbiotas (vaginal, cervical, endometrial, peritoneal fluid, endometriotic lesion, and/or gut) identified Gram-negative bacterial taxa that were significantly enriched in people with endometriosis, which may provide additional support for a putative link between bacterial endotoxins/LPS (part of the outer membrane of Gram-negative bacteria) and endometriosis as proposed by the bacterial contamination hypothesis (reviewed in^38,39^). Similar to Chen et al., 2017 who used endometrial swabs to profile uterine bacteria by 16S rRNA sequencing, none of the endometrial microbiotas profiled in our study were *Lactobacillus*-dominant (> 90% *Lactobacillus* species)^26^. This contrasts with several other studies^25,27–29,31,33,51^ where they report endometrial microbiotas that are *Lactobacillus*-dominant. Instead, the major taxa represented in our study population were *Mycoplasma* (Gram-negative), ‘Others’, *Lactobacillus* (Gram-positive), *Lachnospiraceae* (other) (Gram-positive), *Allobaculum* (Gram-positive), *Moraxella* (Gram-negative), *Bacteroidales* (Gram-negative), *Lachnospiraceae* (Gram-positive), *Lactococcus* (Gram-positive), *Bifidobacterim* (Gram-positive), and *Escherichia* (Gram-negative). We suspect this could be a result of using different extraction methods and biological materials for 16S rRNA sequencing (endometrial fluid^25,29,52^; embryo transfer catheter tip^24,27^; uterine washings^51^) and different populations of patients (fertility patients^25,27–29,51^). In fact, it was recently reported that the microbiota recovered from endometrial tissue samples was not fully reflected in paired endometrial fluid^53^, supporting the notion that different biological materials from the same anatomical location can yield different microbiota compositions. Furthermore, we did not find differences in demographics between our cases and controls, except that symptomatic controls had a significantly earlier age at menarche. It is also important to note that three endometrial biopsies (2 Cases, and 1 Symptomatic Control) did not yield a PCR product for the 16S rRNA gene, and were thus considered negative. This may suggest that not everyone harbours an endometrial microbiota.

The bacteria in the human microbiotas co-evolved with their hosts. Much of our knowledge of bacterial–host interactions has been gleaned from the high diversity gut microbiota, which is critical in modulating host immunity (reviewed in^54^). However, the female reproductive tract microbiotas (vaginal, cervical, endometrial) are lower in diversity^26^ and distinct from the gut^55^. Although the role of the endometrial microbiota in human health and disease is largely unknown, it is becoming increasingly clear that perturbations in endometrial bacteria are associated with pathologies like endometriosis^26,33,51,56^, endometrial cancer^35,36^, uterine fibroids^30^, and in success or failure of pregnancy following IVF^21–23,25^. Further, perturbations in bacterial populations are not limited to the endometrium of people with endometriosis, but may be more widespread (reviewed in^40^). Differences in the vaginal^26,57^, cervical^26,51,57–59^, and gut microbiotas^60,61^ are reported between people with endometriosis and controls in some studies, but not all^62^. Similar to our observations in the endometrial microbiota, two independent reports found increased diversity in the cervical microbiota of people with endometriosis compared to controls^51,59^, further supporting a link between bacterial diversity in the reproductive microbiotas and endometriosis. Interestingly, the relationship between bacterial diversity and endometriosis is opposite in the gut, where individuals with endometriosis had lower bacterial diversity than controls^60,61^. Typically reproductive ‘health’ tends to be associated with a low diversity, *Lactobacillus* dominant vaginal microbiota (reviewed in^63^), while gut ‘health’ tends to be associated with a diverse microbiota (reviewed in^64^). Gut microbiota perturbations have been postulated to contribute to pathogenesis of endometriosis via the regulation of inflammatory processes and estrogen metabolism (reviewed in^65^), and gut permeability is increased in patients with endometriosis^66^ which may contribute to the systemic nature of this condition (reviewed in^67^). Taken together, our observations in the endometrial microbiota, and those of others in the cervix^51,59^ and gut^60^ suggest that people with endometriosis experience dysbiosis, an imbalance of bacteria, at various sites.

Adding to the evidence that microbiota perturbations are linked with endometriosis are several experimental animal studies demonstrating that lesion development^68^, and presence of disease^69–71^ are associated with changes in the gut microbiota of rhesus monkeys and mice. Similar to what Svensson et al., 2021 and Shan et al., 2021 observed in people with endometriosis, Ni et al., 2020 report that mice with experimentally induced endometriosis had lower bacterial diversity and abundance in the gut microbiota than controls^71^. Another piece of evidence of a link between microbiota perturbations and endometriosis is that certain medications (treatment with letrozole, the Traditional Chinese Medicine Shaofu Zhuyu decoction^72^, or antibiotics^73^) reduced endometriotic implant volumes in rats and mice, and was thought to be due in part to restoration of the gut microbiota. However, the challenge in comparing results of microbiota studies in humans and animals is that each study employs different sampling methods and anatomical locations (ie. lower, vs. mid-, vs. upper vagina), sampling timepoints, experimental animals and endometriosis models, 16S rRNA gene sequencing methodologies, and methods of data analysis. Further, from the current literature, it remains unclear which direction the association between bacteria and endometriosis goes; dysbiosis leading to endometriosis, or endometriosis leading to dysbiosis. Nevertheless, increasing evidence suggests perturbations in the microbiotas are associated with endometriosis.

Following reports of bacterial endotoxin in the pelvic cavity and menstrual blood^37^, and “sub-clinical uterine infections” found in patients with endometriosis^56^, Khan et al., 2017 proposed that microbes might activate inflammatory cascades by binding Toll-like receptors, and contribute to endometriotic lesion establishment, growth and progression. This has become known as the ‘bacterial contamination hypothesis’^39^. In addition to the aforementioned microbiota studies, epidemiological studies also support this hypothesis. A large population-based study found females with a history of lower genital tract infections were at a 2.01 times higher risk of endometriosis than those without this history^74^, while another population-based retrospective cohort found a 3.02 times greater risk of being diagnosed with endometriosis in females who had previously had pelvic inflammatory disease (pathogenic bacteria spreading from vagina to upper genital tract)^75^. Furthermore, the association between upper genital tract and peritoneal infections and endometriosis was the focus of a recent systematic review^76^. Although the majority of studies on the microbiotas of people with endometriosis focus on the vaginal, cervical, uterine, or gut microbiotas, studies have profiled the bacteria found in endometriotic lesions^31,77^ and extracellular vesicles (ECVs) isolated from peritoneal fluid of patients with endometriomas^78^ by 16S sequencing, demonstrating that bacteria can be found in disease lesions and ECVs. Hernandes et al., 2020 compared bacteria recovered from the vaginal fluid, eutopic, and ectopic tissues, and reported similar bacterial profiles (*Lactobacillus*, *Gardnerella*, *Streptococcus* and *Prevotella*) at these sites. They did note that deep lesions had altered bacterial profiles (less *Lactobacillus*, more *Alishewanella*, *Enterococcus* and *Pseudomonas*), suggesting that different lesions may support different bacterial populations^31^. Khan et al., 2016 demonstrated that although bacteria could not be cultured from endometriomas (or other non-endometrioma cysts in controls) there was significantly more *Streptococcaceae* and *Staphylococaceae* and less *Lactobacillacae* in endometriomas as compared to non-endometrioma cysts from controls using 16S rRNA sequencing^77^. Although some differences in bacteria found in endometriotic lesions have been reported, pathogenic viruses do not follow the same trend^79^. Vestergaard et al., 2010 quantified 11 common pathogenic DNA viruses in the eutopic endometrium of endometriosis cases and controls, and ectopic lesions, revealing low viral prevalence, no significant differences between cases and controls, and no viruses recovered from ectopic lesions. Combined, these epidemiological studies, and microbiota studies profiling bacteria in the endometriotic lesions support the ‘bacterial contamination hypothesis’, and a role for bacteria in the pathogenesis and/or pathophysiology of endometriosis.

Our study has several strengths including the blinding of researchers to experimental groups until data analysis, the use of 16S rRNA gene sequencing to profile the endometrial microbiota of patients with and without endometriosis, the exclusion of people who were using hormone therapies in the 3 months preceding study enrollment, the inclusion of no template negative controls in each PCR run, and the inclusion of patients with surgically confirmed presence or absence of disease. The limitations of our study include our small sample size, lack of information on antibiotic or probiotic use in the months preceding surgery in our population, inability to explore the function of the endometrial bacteria we recovered, and lack of healthy controls (inclusion of which may have helped determine if there is an endometrial microbiota in “healthy” women). Furthermore, our study did not examine other microbiotas of the uterine ecosystem (e.g. virome, mycome, etc.). Additionally, subsequent studies should include additional negative controls for microbiota library preparation (e.g. extraction controls, procedural swabs, hospital room air swabs, and/or other anatomic locations), and positive controls (e.g. mock bacterial communities). Larger, well-controlled studies aimed at understanding the role of bacteria in the pathogenesis and pathophysiology of endometriosis may offer novel insights into new therapeutics for this chronic condition.

In summary, we report that the endometrial microbiota in the uterus of people with endometriosis is more diverse than that of symptomatic controls (with pelvic pain, but surgical absence of endometriosis). The relative abundance of bacterial taxa enriched in the endometrial microbiota of patients with endometriosis belonged to the Actinobacteria phylum, *Oxalobacteraceae* and *Streptococcaceae* families, and *Tepidimonas* genus, while those enriched in the symptomatic controls belonged to the *Burkholderiaceae* family, and *Ralstonia* genus. Taken together, our study adds to the literature describing perturbations in the endometrial, vaginal, cervical, and gut microbiotas of people with endometriosis.

## Supplementary Information

Supplementary Information.

Supplementary Figure 1.

Supplementary Figure 2.

## Data Availability

Raw sequence reads for data included in this manuscript have been deposited in NCBI’s Gene Expression Omnibus (GEO) under Accession Number GSE172172.
