# Supplementary material for: Endometrial microbiota is more diverse in people with endometriosis than symptomatic controls
Source: Sci Rep. 2021 Sep 23;11:18877. doi: 10.1038/s41598-021-98380-3 (PMC8460742; doi:10.1038/s41598-021-98380-3)
Supplement: Supplementary file 1 — Supplementary Information. [file 41598_2021_98380_MOESM1_ESM.docx]

**Supplemental Information**

**Title:** Endometrial Microbiota is More Diverse in People with Endometriosis than Symptomatic Controls

**Authors:** Jocelyn M. Wessels^a^, Miguel A. Domínguez^b^, Nicholas A. Leyland^a^, Sanjay K. Agarwal^c^, and Warren G. Foster^a*^.

**Affliations:** ^a^Department of Obstetrics & Gynaecology, McMaster University, Hamilton, Ontario, L8S 4K1, Canada; ^b^Facultad de Medicina Veterinaria y Zootecnia, Universidad Autónoma de Tamaulipas, 87000 Cd. Victoria, Tamaulipas, Mexico; ^c^Department of Reproductive Medicine, University of California San Diego, La Jolla, California,

92037, USA.

***Corresponding Author:** Dr. Warren G. Foster, Department of Obstetrics & Gynaecology, McMaster University, Hamilton, Ontario, L8S 4K1, Canada; Phone: (905) 525-9140 ext. 22822; Email: fosterw@mcmaster.ca

**Supplemental Table 1: Participant demographics, gynecological history, and surgical diagnosis.**

| **Characteristic** | **Endometriosis Cases** | **Symptomatic Controls** | **P value** |
| --- | --- | --- | --- |
|  | **n=12** | **n=9** |  |
|  | | | |
| **Age (y), mean±SEM** | **33.8±5.8** | **35.1±3.3** | **0.703** |
|  | | | |
| **Ethnicity, N (%)** |  | | |
| **Caucasian** | **6 (50)** | **4 (44)** | **0.856** |
| **Other** | **2 (17)** | **1 (11)** |  |
| **Unknown** | **4 (33)** | **4 (44)** |  |
|  | | | |
| **Occupational status, N (%)** |  | | |
| **Employed** | **5 (42)** | **2 (22)** | **0.520** |
| **Unemployed** | **1 (8)** | **2 (22)** |  |
| **Unknown** | **6 (50)** | **5 (56)** |  |
|  | | | |
| **Smoking status, N (%)** |  | | |
| **Nonsmoker** | **9 (75)** | **8 (89)** | **0.603** |
| **Smoker** | **3 (25)** | **1 (11)** |  |
|  | | | |
| **Gynecological history** |  | | |
| **Age at menarche (y), mean±SEM** | **13.0±1.3** | **11.6±1.0** | ***0.015*** |
| **Years since menarche (y), mean±SEM** | **20.8±1.6** | **23.6±3.2** | **0.399** |
| **Duration of bleeding (d), mean±SEM** | **5.7±1.9** | **5.7±1.4** | **0.938** |
| **Menstrual cycle stage, N (%)** |  | | |
| **Menstrual** | **0 (0)** | **2 (22)** | **0.315** |
| **Proliferative** | **6 (50)** | **4 (44)** |  |
| **Secretory** | **5 (42)** | **3 (33)** |  |
| **Unknown** | **1 (8)** | **0 (0)** |  |
| **Stage of endometriosis, N (%)** |  |  |  |
| **Minimal, 1** | **0 (0)** | **-** |  |
| **Mild, 2** | **1 (8)** | **-** |  |
| **Moderate, 3** | **1 (8)** | **-** |  |
| **Severe, 4** | **10 (83)** | **-** |  |
| **Control pathologies, N (%)** |  |  |  |
| **Fibroids** | **-** | **1 (11)** |  |
| **Cysts** | **-** | **5 (56)** |  |
| **Other (Ulcerative colitis (n=1), idiopathic pelvic pain (n=2))** | **-** | **3 (33)** |  |

**Supplemental Figure 1: Agarose Gel of 16S PCR Products.**

**(A)** A representative 1.5% agarose gel showing the presence or absence of a PCR product following a two-stage (nested) PCR for the 16S rRNA gene. Samples positive for the 300 base pair (bp) PCR product (see gel lane entitled “16S rRNA positive Endometrial Biopsy”) were sent for sequencing at the McMaster Genomics Facility, on the Illumina miSeq platform as described in Methods. Samples that did not have the 300bp band (see gel lanes entitled “16S rRNA negative Endometrial Biopsy”, “PCR negative 1”, “PCR negative 2”) were not sent for sequencing. Bands on the gel at 80bp represent PCR primer dimers. **(B)** The full-length gel pictured in panel A.

**Supplemental Figure 2: Endometrial microbiota of patients with stage 4 endometriosis is significantly more diverse than symptomatic controls.** Three alpha-diversity metrics were used to compare bacterial richness and evenness within the endometrial microbiota of patients with surgically confirmed stage 4 endometriosis (N=10) and surgically confirmed controls (with pelvic pain, but not endometriosis, N=9). **(A)** No significant differences in Observed Species or **(B)** Chao 1 Richness were observed at the levels of rarefaction where graphs levelled off and the greatest number of samples was retained (multiple unpaired t-tests, corrected for multiple comparisons using the Holm-Sidak method). **(C)** Patients with stage 4 endometriosis had significantly greater bacterial diversity as assessed by the Shannon Diversity Index at all levels of rarefaction where the graph levelled off and the greatest number of samples was retained (adjusted P≤0.01; multiple unpaired t-tests, corrected for multiple comparisons using the Holm-Sidak method). **(D)** The average percent relative abundance of the top 10 taxa in the endometrial microbiota of stage 4 cases and controls was plotted as a pie chart and the proportion of taxa assigned to the ‘Others’ category was significantly greater in patients with stage 4 endometriosis than symptomatic controls (unadjusted P=0.008; adjusted P=0.08, Mann-Whitney U test without/with correction for multiple comparisons using the Holm-Sidak method). ** P≤0.01. Data is presented as mean ± SEM **(A-C)** and as a percentage **(D)**. Ns: not significant. OTUs: operational taxonomic units. ###: resolved to bacterial order, ####: resolved to bacterial family.
