# Supplementary figures and images for: Endometrial microbiota is more diverse in people with endometriosis than symptomatic controls
Source: Sci Rep. 2021 Sep 23;11:18877. doi: 10.1038/s41598-021-98380-3 (PMC8460742; doi:10.1038/s41598-021-98380-3)

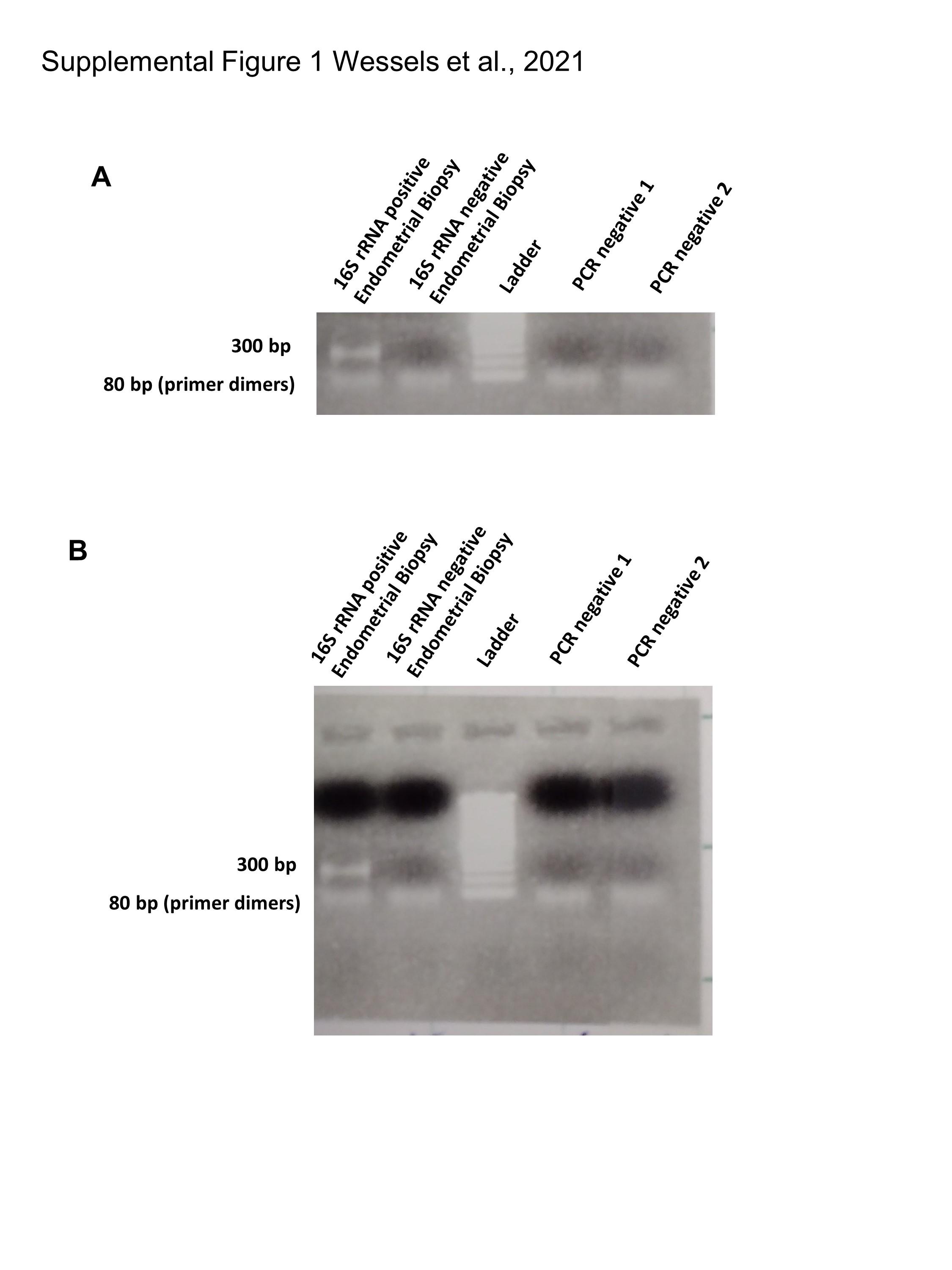

Supplement: Supplementary file 2 — Supplementary Figure 1. [file 41598_2021_98380_MOESM2_ESM.jpg]

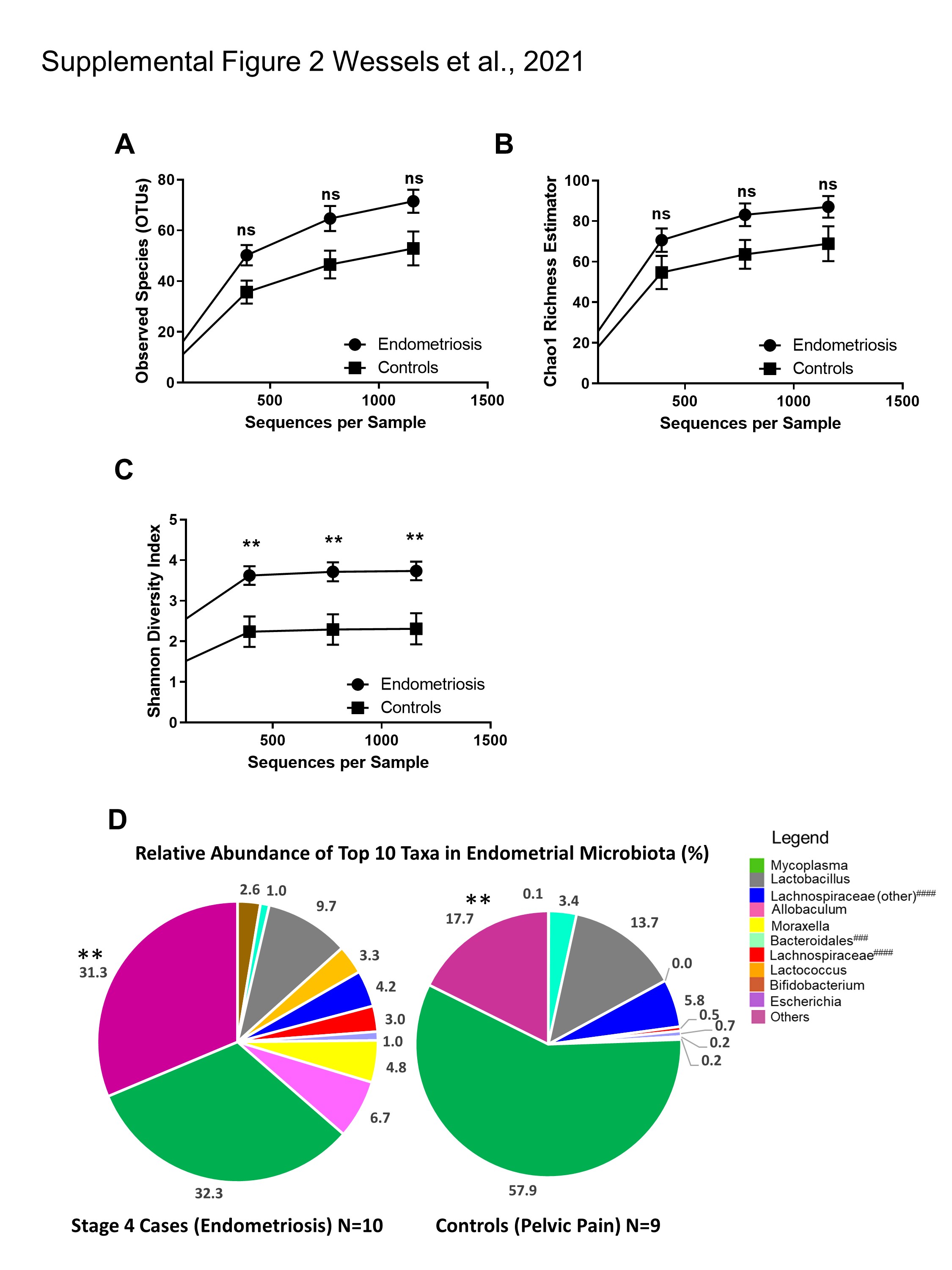

Supplement: Supplementary file 3 — Supplementary Figure 2. [file 41598_2021_98380_MOESM3_ESM.jpg]
